# Minimally Invasive Surgery for ICH Evacuation Combined With Deferoxamine Treatment Increased Perihematomal Claudin-5 and ZO-1 Expression Levels and Decreased BBB Permeability in Rabbits

**DOI:** 10.3389/fneur.2022.835494

**Published:** 2022-03-03

**Authors:** Siying Ren, Shanshan Han, Likun Wang, Yuanxin Huang, Jing Wu, Guofeng Wu

**Affiliations:** ^1^Department of Emergency, The Affiliated Hospital of Guizhou Medical University, Guiyang, China; ^2^Graduate School of Guizhou Medical University, Guiyang, China

**Keywords:** intracerebral hemorrhage, minimally invasive surgery, deferoxamine, tight junction proteins, blood-brain barrier permeability

## Abstract

**Objective:**

To investigate the role of minimally invasive surgery (MIS) in intracerebral hemorrhage (ICH) evacuation combined with deferoxamine (DFX) treatment on perihematomal tight junction protein (claudin-5 and ZO-1) expression levels and blood-brain barrier (BBB) permeability in rabbits.

**Methods:**

We randomly assigned 65 male rabbits (weight: 1.9–2.6 kg) to a normal control group (NC group, 13 rabbits), hemorrhage model group (HM group, 13), DFX treatment group (DFX group, 13 rabbits), MIS group (MIS group, 13 rabbits), or MIS combined with DFX treatment group (MIS + DFX group, 13 rabbits). ICH was established in all of the groups except the NC group. MIS was performed to evacuate the hematoma 6 h after the ICH model was created in the MIS and MIS + DFX groups. The DFX and MIS + DFX groups were treated with DFX (100 mg/kg, dissolved in 2 mL of 0.9% saline solution, administered intramuscularly) at 2 h, and then every 12 h for 7 d. The same dose of 0.9% saline solution was administered to the NC, HM, and MIS groups at the same time points. Sixty-five rabbits were divided into 5 groups, and 13 rabbits in each group. Neurological deficit (i.e., Purdy's score) was recorded in all rabbits before euthanasia (N total = 65). In each group, 2 rabbits were used for iron concentration measurement (N total = 10), 2 rabbits were used for brain water content measurement (N total = 10), 3 rabbits were used for BBB permeability measurement (N total = 15), 3 rabbits were used for claudin-5, ZO-1 expression detection by Western Blotting (N total = 15), and 3 rabbits were used for claudin-5, ZO-1 mRNA detection by real-time PCR (N total = 15). On day 7, the rabbits were sacrificed and the perihematomal brain tissue was harvested to test the iron concentration, brain water content (BWC), tight junction proteins (claudin-5 and ZO-1) expression, and BBB permeability.

**Results:**

Purdy's score, iron concentration, and BWC were lower in the MIS and MIS + DFX groups compared to the HM and DFX groups. The MIS + DFX group showed a significant decrease in these indicators. The use of MIS to evacuate the hematoma led to increased expression levels of claudin-5 and ZO-1, as well as decreased BBB permeability. The MIS + DFX group exhibited a remarkable increase in claudin-5 and ZO-1 expression levels and a significant decrease in BBB permeability.

**Conclusions:**

MIS combined with DFX treatment could increase the expression levels of perihematomal tight junction proteins (claudin-5 and ZO-1) expression, reduce BBB permeability, and improve the neurological function. MIS combined with DFX treatment may also prevent secondary brain damage following ICH.

## Introduction

Intracerebral hemorrhage (ICH) has the highest mortality rate of any type of stroke; 46% of patients die or still have severe disability 1 year after the ICH. ICH is the most common cause of death and disability among Chinese residents. The severe impact on patients and society has made China the country with the heaviest burden of ICH worldwide (1–3). Nevertheless, there are no effective treatments for ICH, the clinical outcome remains poor and many challenges remain (4). Craniotomy for ICH evacuation is an aggressive treatment option that may lead to iatrogenic injury in some patients. A recent randomized clinical trial from China reported that a minimally invasive craniopuncture technique can improve the independent survival rates of ICH patients compared to conservative treatment. Minimally invasive craniopuncture appears safe and effective for ICH treatment (5, 6).

After ICH onset, red blood cells (RBCs) infiltrate the brain tissue and continuously lyse to release hemoglobin. Hemoglobin/iron deposit-induced oxidative damage leads to neuronal ferroptosis and poor neurological outcomes. Iron, a heme degradation product, plays an important role in ICH-induced brain injury. RBC disintegration leads to brain iron overload following ICH. Iron overload is closely related to poor outcome in ICH patients (7–9). Peroxidation catalyzed by iron is an important cause of brain damage. Iron damages the endotheliocytes and pericytes, which constitute the blood brain barrier (BBB), thereby degrading tight junction (TJ) proteins (claudin-5 and ZO-1), destroying the BBB integrity, and causing secondary brain injury (10).

Minimally invasive surgery (MIS) is an effective treatment option that may have superior benefit for ICH patients compared to other treatment options (11, 12). However, MIS does remove all RBCs, iron, and other neurotoxic substances, which extravasate into the perihematomal brain tissue (13–16). MIS alleviates secondary brain damage, but has limitations. Hematoma evacuation with MIS combined with the use of medications may alleviate secondary brain injury during ICH treatment (13, 17).

DFX, an iron chelator, rapidly penetrates the BBB and accumulates in the brain tissue at a significant concentration after intramuscular or subcutaneous injections (18, 19). DFX can prevent damage caused by iron overload and iron-mediated toxicity after ICH (20). DFX treatment reduces iron deposition and brain edema, and improves neurologic outcomes after ICH (21–23). Similar to MIS, DFX use also has limitations. In particular, DFX treatment did not improve outcomes in a collagenase-induced ICH rat model compared to the use of a whole-blood model (24). To explore the effect of DFX treatment in ICH and determine whether MIS combined with DFX treatment may be appropriate for ICH treatment, we evaluated the effect of MIS in hematoma evacuation combined with intramuscular DFX treatment on secondary brain damage in an ICH rabbit model.

## Materials

### Main Reagents

Urethane (Hefei BASF Bio-technology Co., Ltd., Anhui, China), penicillin (Harbin Pharmaceutical Group, Harbin, China), urokinase (Wuhan Renfu Pharmaceutical Co., Ltd., Wuhan, China), deferroamine mesylate (Novartis Pharma GmbH, Weil, Germany), iron determination kit (Nanjing Jiancheng Institute of Bioengineering, Nanjing, China), 4% paraformaldehyde (Sinopharm Chemical Reagents Co., Ltd., Shanghai, China), PBS phosphate buffer (Beijing Zhongshan Jinqiao Biotechnology Co., Ltd., Beijing, China), claudin-5, ZO-1 rabbit polyclonal antibody (Boolsen Biotechnology Co., Ltd., Beijing, China), and PrimeScript™ RT reagent Kit with gDNA Eraser and HiScript II One Step QRT-PCR SYBR Green Kit (Nanjing Novus Biotechnology Co., Ltd., Nanjing, China) were used for the experiments. The following reagents were purchased from Kangwei Century Company, Beijing, China: Protease inhibitor, BCA protein quantitative Kit, WB (HRP) Kit (rabbit), WB (HRP) Kit (mouse), Ultrapure RNA Kit, Goat anti-rabbit. claudin-5 and ZO-1 (Boolsen Biotechnology Co., Ltd., Beijing, China), GAPDH and β-actin (Jing Tiancheng Biotechnology Co., Ltd., Beijing, China), formamide (Shanghai Aladdin Biochemical Technology Co., Ltd., Shanghai, China), and Evans Blue (Beijing Solebo Technology Co., Ltd., Beijing, China) were purchased from the respective manufacturers.

### Main Instruments

A ZH-Lanxing B-Type rabbit stereotaxic apparatus (Huaibei Zhenghua Biological Instrument & Equipment Co. Huaibei, Anhui, China), UV-visible Spectrophotometer (Beijing Universal Analysis Instrument Co., Ltd., Beijing, China), Real-time Fluorescence Quantifier (RT-PCR) (Step One Plus TM, Applied Biosystems, Foster City, CA, USA), low-temperature centrifuge (Hunan Xiangyi Experimental Instrument Factory, Hunan, China), electrophoresis apparatus (Shanghai Tieneng Technology Co., Ltd., Shanghai, China), electronic balance (Shimadzu Co., Ltd., Kyoto, Japan), and a constant temperature drying oven (Tianjin Tester Co., Ltd., Tianjin, China) were used for the experiments.

## Methods

### Experimental Groups

The study protocols were approved by the Animal Care and Use Committee of Guizhou Medical University, China, and performed according to the criteria of satisfactory laboratory practice for drugs. A total of 65 rabbits (1.9–2.6 kg) were provided by the Experimental Animal Center of Guizhou Medical University. The rabbits were kept at 10–25°C by a special animal breeder. Rabbits were fed animal fodder and water. Rabbits were randomly divided into a normal control group (NC group, 13 rabbits), a hemorrhage model group (HM group, 13 rabbits), a DFX medication group (DFX group, 13 rabbits), a MIS group (MIS group, 13 rabbits), and a MIS combined with DFX treatment group (MIS + DFX group, 13 rabbits). ICH was induced in all of the groups except NC group. The rabbits were sacrificed under anesthesia on day 7 after the relevant treatment.

### ICH Model Preparation

The methods used in this study for establishing the ICH model were similar to those used in our previous studies (25, 26). First, rabbits were anesthetized using injections of 20% urethane (2 mL/kg) into the marginal ear vein. The anesthetized rabbit was fastened to a stereotaxic apparatus, and the skin in the operative area was disinfected with povidone iodine. The skin was incised (3 cm) to expose the bregma and lambdoid demarcations. The head was adjusted to position the bregma to be 1.5 mm higher than the lambdoid demarcation. The bregma cross-suture junction was used as a reference point; the puncture point was selected 6 mm to the left along the coronal plane and 1 mm parallel to the sagittal plane. The skull was drilled using a dental drill (1 mm in diameter) at the puncture point, and 0.5 mL of autologous arterial blood was extracted from the central ear artery using an insulin syringe. The syringe was connected to a size-7^#^ needle with a flat tip. The size-7^#^ needle was quickly inserted vertically into the puncture point in the skull up to a depth of 12 mm, and 0.3 mL of autologous arterial blood (similar to a 30 mL basal ganglia hematoma in humans) was slowly injected into the basal ganglia. The injection lasted for at least 3 min. The needle was retained in the same position for 8 min after injection, followed by slow removal of the needle. The drill hole was sealed with bone wax to prevent pneumocephalus.

The rabbits were sent back to the experimental animal center and fed as usual for 7 d. Animals received an intramuscular injection of penicillin (400,000 U) once daily for 3 d to prevent infection.

**Figure 1:**
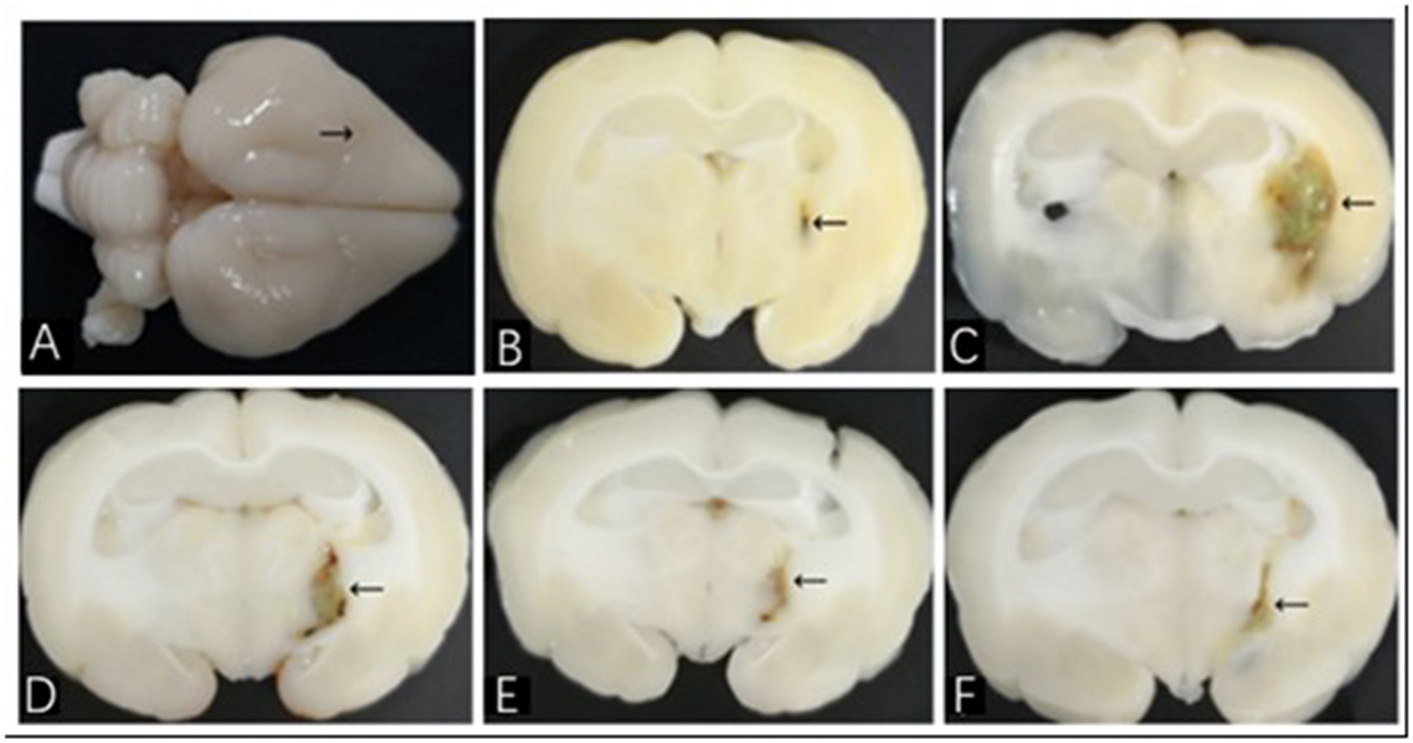
Brain histological sections of ICH model in rabbit. **(A)** The whole brain specimen of rabbit; the arrow shows the puncture point; **(B–F)** show histological sections of NC, HM, DFX, MIS, and MIS + DFX groups, respectively. The arrows show hematoma.

All of the rabbits were underwent pathological analysis of brain tissue at day 7. Neurological deficit scores >2 (recorded before euthanasia) or basal ganglia hematoma, with no empirical evidence of damage or lateral ventricle hematoma, was required for the ICH model to succeed (Figure 1C). Exclusion criteria during and after MIS included backflow along the needle track, blood in the ventricle, and death.

### MIS Procedures

According to our previous studies, the optimal time of MIS is 6–12 h after ICH (27). MIS was performed to evacuate the hematoma 6 h after the ICH model was established. The rabbits were anesthetized again and placed in the stereotaxic apparatus. A size-7# needle with a flat tip was inserted into the hematoma along the same drill hole, and an insulin syringe was connected to the needle; 0.1 mL/5000 U of urokinase (urokinase 100,000 U dissolved in 2 mL of 0.9% saline solution; 0.1 mL = 5000 U) was injected into the hematoma. The needle was kept inside the hematoma for 1 h, followed by withdrawal of the needle while slowly aspirating the hematoma. The skin was disinfected and sutured. The rabbits in the NC group were treated using the same procedures (i.e., 0.3 mL of 0.9% saline solution was injected into the puncture region, and 0.1 mL of 0.9% saline solution was infused into the same area again at 6 h). In the HM and DFX groups, a sham MIS was performed 6 h after the ICH model was established, by infusing 0.1 mL of 0.9% saline solution into the hematoma at 6 h. The rabbits in the DFX group were given the DFX solution intramuscularly (100 mg/kg, dissolved in 2 mL of 0.9% saline solution) at 2 h, followed by repeat administration every 12 h for 7 d. In the MIS+DFX group, MIS was used to evacuate the hematoma, followed by intramuscular injection of the same amount of DFX solution. The rabbits were sacrificed on day 7 after the corresponding procedures. Histopathological analysis was performed to evaluate the effectiveness of MIS (Figure 1E).

### Brain Tissue Preparation

The methods used for preparation of brain tissue were the same as those used in our previous studies (25, 26). We injected 20% urethane (2 mL/kg) to anesthetize the rabbits. The brain was placed on ice. With the hematoma placed at the center, brain tissues around the hematoma were sliced and divided into four parts: anterior, posterior, left, and right. A total of 5 mm of brain tissues surrounding the hematoma were harvested from each part to measure the iron content, brain water content (BWC), expression levels of claudin-5 and ZO-1, and BBB permeability.

### Neurological Deficit Score (Purdy's Score) Evaluation

Neurological deficit was recorded after the rabbit awoke from anesthesia and 2 h before euthanasia on day 7 after the corresponding treatments were performed. A neurological deficit scale (Purdy's score) (28) was used to compare the neurological function among the experimental groups. Tests were performed by two researchers who were blinded to the treatments. The tests included an evaluation of the motor function (score of 1–4), conscious level (score of 1–4), head turning (score of 0–1), circling (score of 0–1), and hemianopsia (score of 0–1). A score of 11 indicated maximum impairment (comatose or death), whereas a score of 2 indicated normal examination.

### Iron Concentration Measurement

The double steaming water colorimetry method was used to measure perihematomal iron concentration. The perihematomal brain tissues were weighed to determine the iron concentration [weight (g): volume (mL) = 1:9]. Therefore, saline was added at a volume 9 times the weight of the brain tissue. Mechanical homogenization was performed under ice-water bath condition; the homogenate was centrifuged for 10 min at 2,500 rpm. The supernatant was obtained and tested to determine the iron concentration using the Iron Determination Kit (Nanjing Jiancheng Institute of Bioengineering, Nanjing, China).

### BWC Measurement

The dry- and wet-weight method was used to measure the BWC. Brain tissues surrounding the hematoma were used to measure the BWC. First, the wet tissues were weighed, and the samples were placed in an oven at 100°C for 48 h. The dried samples were also weighed (with an accuracy of 0.1 μg). BWC was calculated as follows: (wet weight**—**dry weight)/wet weight × 100%.

### BBB Permeability Measurement

Evens Blue (EB) was applied as a tracer to estimate the BBB permeability. Two h before brain harvesting, EB solution (2 mL/kg) was injected into the ear vein of the rabbits. Brain tissues surrounding the hematoma were quickly obtained and weighed on an electronic balance (with an accuracy of 0.1 mg). The samples were placed in a test tube with 4 mL of formamide. The test tube was capped and placed into a 54°C constant-temperature water bath for 24 h for EB staining. The test tube was centrifuged at 2,400 rpm for 5 min. The supernatant was absorbed using a pipette, and the absorbance was measured using a spectrophotometer (λ = 632 nm). Formamide solution was applied as a blank control. EB content was measured from the standard curve, as described in our previous studies (25, 26). The formula used was as follows: EB content in brain tissues (μg/g wet brain) = B × formamide (mL)/wet weight (g); where B is the sample EB content (μg/mL) obtained from the linear regression equation base of the standard curve.

### Real-Time PCR for Claudin-5, ZO-1 mRNA Detection

Brain tissues surrounding the hematoma were obtained. An electronic balance (~30 mg, with an accuracy of 0.1 mg) was used to weigh the brain tissues. The brain tissues were pulverized, and total RNA was separated using a Trizol Reagent box. Total RNA was used to produce cDNA using a PrimeScript™ RT reagent Kit with gDNA Eraser. Data were normalized to those of GAPDH. Primer sequences used for claudin-5, ZO-1, and GAPDH are listed in Table 1.

**Table 1:** Sequences for primers.

**Gene name**		**Primer** **sequences**	**Product (bp)**
claudin-5	Forward	5′-TCCAGTGCAAAGTCTTCGAC-3′	243
	reverse	5′-TGTTGCCATACCATGCTGTG-3′	
ZO-1	Forward	5′-AGGGGCAGCTACAGGAAAAT-3′	173
	reverse	5′-TGGTTCAGGATCAGGACGAC-3′	
GAPDH	Forward	5′-CATGTTTGTGATGGGCGTGA-3′	244
	reverse	5′-GGAGGCAGGGATGATGTTCT-3′	

### Western Blotting for Claudin-5, ZO-1 Expression Detection

Brain samples were homogenized and centrifuged to determine the protein concentrations. Proteins were separated using 10% sodium dodecyl sulfonate- polyacrylamide gel (SDS-PAGE) electrophoresis and transferred to a nitrocellulose film. The protein concentrations were determined by marking and using one-step fast WB kit (HRP). Antibodies used for incubation were as follows: primary antibody β-actin 1:500, goat anti claudin-5 polyclonal antibody 1:100, mouse anti ZO-1 monoclonal antibody 1:500, and secondary antibody 1:400. The antibodies were incubated overnight at 4°C and rinsed for chemiluminescence, followed by film exposure. Gel-Pro analyzer 4 image analysis software was used to measure the gray values of each band, and the results were expressed as the ratio of integral optical density value for claudin-5/β-actin and ZO-1/β-actin.

### Statistical Analysis

SPSS software (version 19.0; IBM Corp., Armonk, NY, USA) was used for statistical analysis. Data are presented as mean ± standard deviation (X ± SD). ANOVA was used to compare the groups. The groups were compared using the Fisher's (F) test when the variances were equal. An F test (Welch test) was used when the variances were not equal. Multiple comparisons with the means were checked using Dunnett's T3 (unequal variances) tests. *P-*values < 0.05 were considered to be statistically significant.

## Results

### ICH Model Preparation

After the injection of autologous arterial blood into the basal ganglia, the rabbits manifested with contralateral hemiplegia and were unable to walk or crawl. Neurological deficit scores > 2 demonstrated that the ICH model in this study was successful. A total of 72 rabbits were used in this study, of which 65 underwent the ICH model successfully according to the experimental requirements. Seven rabbits died accidentally. The brains of dead rabbits were dissected and examined, and results showed that two rabbits in the MIS group had died of intracranial infection. One rabbit died due to overdose of anesthetic agents in the NC group. Two rabbits in the MIS+DFX group died of unclear causes. One rabbit in the HM group died of status epilepticus, and one died of lung infection. These seven rabbits were excluded from the study. The other 65 rabbits were included in the experiments. There were 13 rabbits in each group. All of the rabbits tolerated ICH and MIS. They survived until the experiment was terminated.

### Brain Histological Sections of the ICH Model in Rabbits

The rabbits were sacrificed on day 7 after the corresponding interventions were performed. The whole brain was observed with the naked eyes (Figure 1A), and the brain was sliced along the coronal puncture point. Analysis of the brain tissues from the NC group revealed clearly discernible, bilaterally symmetrical brain structure; a slight injury focused on the puncture point (Figure 1B). In the HM group, an oval hematoma was observed along with brownish-yellow margins in the left basal ganglia. The midline structures were not displaced and there was severe edema around the hematoma (Figure 1C). Compared with the HM group, the hematoma volume in the DFX group (Figure 1D), MIS group (Figure 1E), and MIS + DFX group (Figure 1F) were smaller and there was less hemosiderin deposition around the hematoma. The MIS + DFX group exhibited excellent results compared to the DFX and MIS groups.

### Neurological Deficit Score (Purdy's Score) Changes

**Figure 2:**
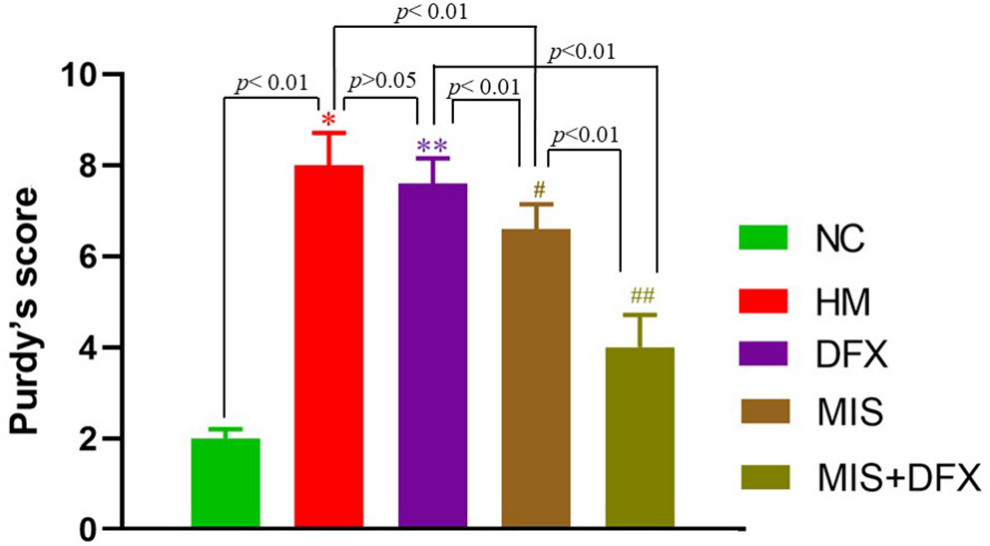
Changes in neurological deficit (Purdy's score). MIS combined with DFX treatment was superior to both MIS and DFX treatment alone. Evaluating the neurological deficit by Purdy's score. *p < 0.01 vs. NC group; **p > 0.05 vs. HM group; ^#^*p* < 0.01 vs. HM and DFX groups; ^##^*p* < 0.01 vs. DFX and MIS groups. Data are presented as mean ± SD.

The neurological function scores were significantly higher in the HM group compared to the NC group (*p* < 0.01), suggesting that ICH was associated with impaired neurological function. There was no significant difference in neurological deficit between the DFX and HM groups (*p* > 0.05). The MIS group exhibited lower neurological function scores compared to those in the HM and DFX groups (*p* < 0.01). Following MIS combined with DFX treatment, the neurological function scores were significantly decreased compared to those in the DFX or MIS group (*p* < 0.01), suggesting that MIS combined with DFX treatment was superior to only MIS or DFX treatment (Figure 2).

### Perihematomal Iron Concentration

**Figure 3:**
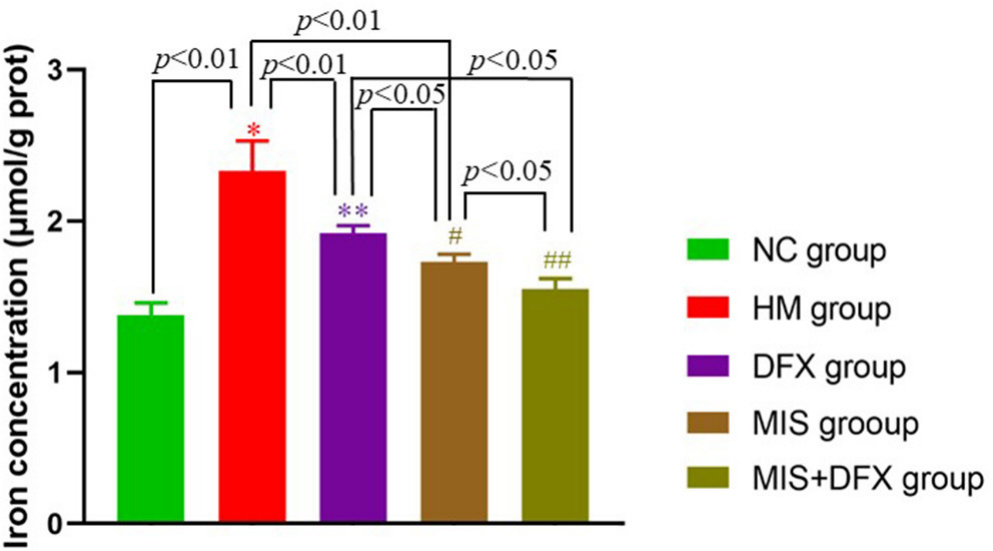
Changes in perihematomal iron concentration. MIS combined with DFX treatment was found to reduce iron overload surrounding hematoma. Testing the iron concentration by double steaming water colorimetry. *p < 0.01 vs. NC group; **p < 0.01 vs. HM group; ^#^*p* < 0.01 vs. HM group; ^#^*p* < 0.05 vs. DFX group; ^##^*p* < 0.05 vs. DFX and MIS groups. Data are presented as mean ± SD.

Iron concentration around the hematoma in the HM group was significantly higher than that in the NC group (*p* < 0.01), suggesting that hemoglobin was released due to RBC lysis after ICH. In the MIS and DFX groups, iron concentration was decreased compared to that in the HM group (*p* < 0.01), suggesting that both MIS and DFX treatments could decrease the iron concentration. Additionally, MIS combined with DFX treatment significantly decreased the iron concentration compared to that in the MIS and DFX groups (*p* < 0.05) (Figure 3).

### Perihematomal BWC

**Figure 4:**
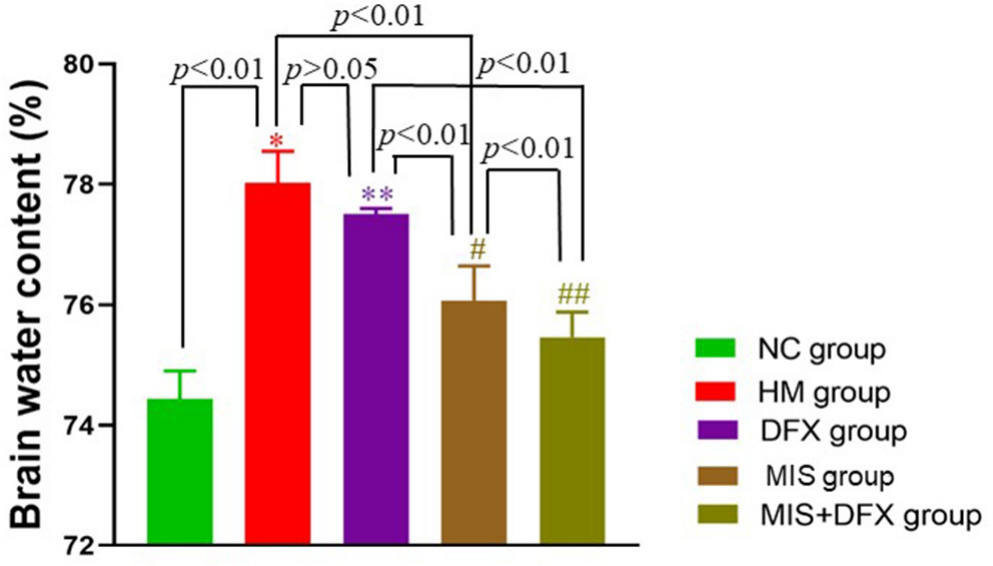
Changes in perihematomal BWC. MIS combined with DFX treatment was found to decrease perihematomal BWC. Evaluating BWC by dry- and wet-weight method. *p < 0.01 vs. NC group; **p > 0.05 vs. HM group; ^#^*p* < 0.01 vs. HM and DFX groups; ^##^*p* < 0.01 vs. DFX and MIS groups. Data are presented as mean ± SD.

BWC in the HM group was significantly higher compared to that in the NC group (*p* < 0.01), suggesting that ICH-induced impaired BBB permeability resulted in brain edema. There was no significant difference in the BWC between the DFX and HM groups (*p* > 0.05). The MIS group had decreased BWC compared to that in the HM group (*p* < 0.01). Following MIS combined with DFX treatment, the BWC was significantly decreased compared to those in the DFX and MIS groups (*p* < 0.01), suggesting that MIS combined with DFX therapy was superior to MIS or DFX treatment alone (Figure 4).

### Perihematomal BBB Permeability

**Figure 5:**
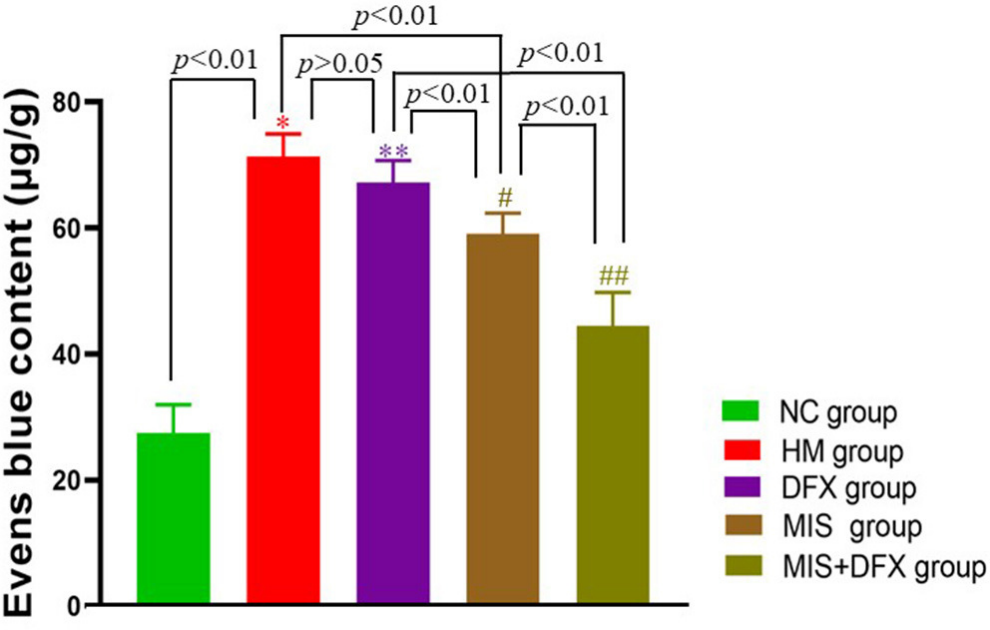
Changes in perihematomal BBB permeability. MIS combined with DFX treatment was found to decrease perihematomal BBB permeability. Evens Blue was applied as a tracer to estimate the BBB permeability. *p < 0.01 vs. NC group; **p > 0.05 vs. HM group; ^#^*p* < 0.01 vs. HM and DFX groups; ^##^*p*<0.01 vs. DFX and MIS groups. Data are presented as mean ± SD.

The perihematomal EB content in the HM group was significantly higher compared to that in the NC group (*p* < 0.01), suggesting that the BBB was severely damaged after ICH. Although there was no significant difference in perihematomal BBB permeability between the DFX and HM groups (*p* > 0.05), the MIS group showed lower BBB permeability compared to that in the HM group (*p* < 0.01). The MIS + DFX group had superior outcomes compared to those in the MIS and DFX groups (*p* < 0.01). These results demonstrated that MIS to evacuate the ICH reduced the ICH-induced BBB damage, and MIS combined with DFX treatment was superior to decrease BBB permeability (Figure 5).

### Perihematomal Claudin-5 and ZO-1 Proteins

**Figure 6:**
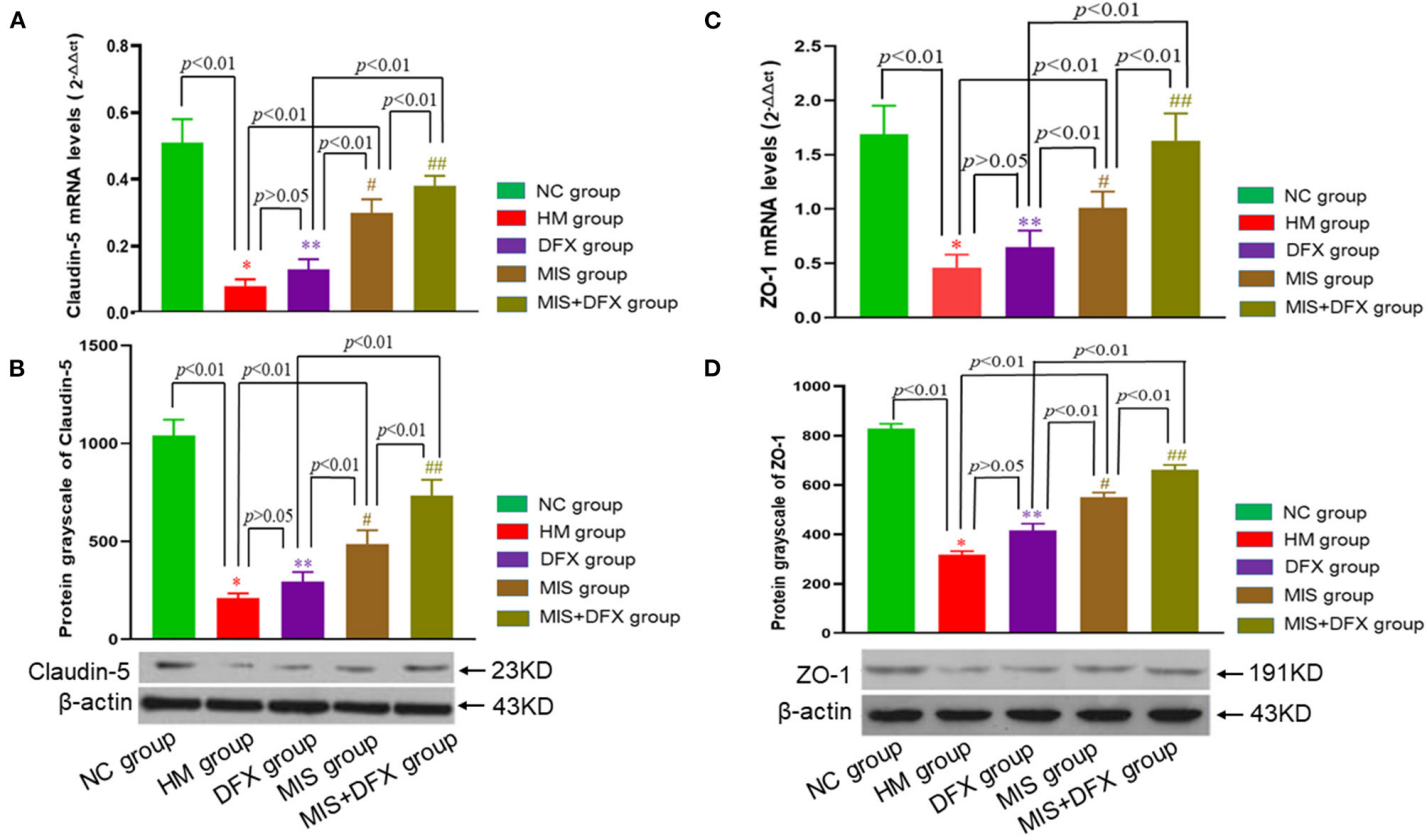
Changes in perihematomal claudin-5 and ZO-1 expression levels. Real-time PCR and Western blotting were used to determine the claudin-5 and ZO-1 expression levels. MIS combined with DFX therapy was found to increase perihematomal claudin-5 and ZO-1 expression levels. **(A,C)** Claudin-5 mRNA and ZO-1 mRNA levels. *p < 0.01 vs. NC group; **p > 0.05 vs. HM group; ^#^*p* < 0.01 vs. HM and DFX groups; ^##^*p* < 0.01 vs. DFX and MIS groups. **(B,D)** Claudin-5 and ZO-1 protein expression levels. *p < 0.01 vs. NC group; **p > 0.05 vs. HM group; ^#^*p* < 0.01 vs. HM and DFX groups; ^##^*p* < 0.01 vs. DFX and MIS groups. Data are presented as mean ± SD.

Real-time PCR and western blotting were used to determine the TJ proteins claudin-5 and ZO-1 levels. Claudin-5 and ZO-1 mRNA and protein expression levels were significantly lower in the HM group compared to the NC group, suggesting that the BBB was damaged due to the ICH. Although there was no significant difference in claudin-5 or ZO-1 levels between the DFX and HM groups (*p* > 0.05), claudin-5 and ZO-1 mRNA and protein expression levels were higher in the MIS group than in the HM and DFX groups (*p* < 0.01). The MIS group was superior to the DFX treatment group. The MIS+DFX group had better results compared to the MIS group or DFX group (*p* < 0.01). These outcomes suggested that MIS to evacuate the hematoma increased the TJ protein levels (claudin-5 and ZO-1). MIS was superior to DFX therapy alone and MIS combined with DFX treatment was most effective for ICH-induced BBB damage (Figure 6).

## Discussion

ICH is a stroke sub-type and represents a major public health problem worldwide. ICH is associated with poor outcomes and high morbidity, disability, and mortality (29). There are no effective treatment options for ICH (30). In recent years, MIS has been used as an alternative to craniotomy due to its improved survival rate and lower complication rate (31). Several experimental studies have reported that MIS is effective for ICH treatment (25, 32). Although MIS can remove most of the hematoma, thereby reducing its size and alleviating the mechanical brain injury due to compression, neurotoxic substances (e.g., thrombin, hemoglobin, and iron) are not removed because they are extravasated into the surrounding brain tissues. Therefore, the effect of MIS on secondary brain injury is limited.

Brain edema, BBB destruction, and neuronal death are manifestations of secondary brain injury, which may be observed in the perihematomal region. ICH-induced BBB disruption is a key pathophysiological process in brain injury (33). The TJ protein (claudin-5 and ZO-1) expression levels are associated with BBB integrity and are major biomarkers of ICH-induced brain injury (34). Hemoglobin, heme, and iron are released after RBC lysis, which aggravate ICH-induced BBB destruction (35). Iron damages the endotheliocytes and pericytes, destroying the BBB integrity (10). Therefore, MIS for hematoma evacuation followed by treatment with the iron chelator DFX may prevent secondary brain injury.

In the HM group of our study, the hematoma-occupying effects persisted and the neurotoxic substances extravasated into the perihematomal brain tissues, which manifested as iron overload, reduced expression levels of claudin-5 and ZO-1, severe BBB disruption, brain edema, and increased neurological function scores compared to the NC group. These results suggest that after ICH, iron is released from the lysed RBCs, which damages the BBB integrity, leading to secondary brain injury and neurological dysfunction.

In the DFX group, although the iron concentration was significantly decreased, the BWC and BBB permeability were only slightly decreased, and neurological function showed slight improvement. There were no significant differences between the HM and DFX groups. These results suggest that DFX use alone does not significantly improve the neurological outcome.

In the MIS group, MIS to evacuate the hematoma reduced the mass effect and prevented the release of iron and other neurotoxic substances from the RBCs, which manifested as decreased iron content, increased claudin-5 and ZO-1 expression levels, reduced BBB permeability, improved brain edema and neurological function compared to the HM group. These results indicated that MIS could alleviate secondary brain injury after ICH. Previous studies reported that MIS significantly reduced the damage to the internal capsule fibers and improved neurological function in a dog ICH model (36). Moreover, MIS effectively reduced matrix metalloproteinase-9 (MMP-9) expression and BBB permeability (32). A meta-analysis showed that MIS treatment improved the outcome in ICH patients compared to the conservative treatment (37). Our results were consistent with those of previous studies.

Additionally, some studies found that DFX treatment significantly reduced iron overload after ICH, but it did not improve the outcome in a collagenase-induced ICH rat model (24, 38). A recent multicenter, randomized, placebo-controlled, double-blind phase 2 trial showed that DFX treatment was safe for ICH patients, but it was not associated with a favorable clinical outcome (i.e., modified Rankin Scale score of 0–2) at day 90 (39). These results were similar to those of our study. DFX use only significantly decreased iron accumulation, but it did not significantly improve neurological function. The reasons may be related to the delayed removal of the hematoma and to the continuous release of neurotoxic substances. Therefore, combined treatment may be the optimal choice for ICH patients.

In the MIS + DFX group, iron concentration, BBB permeability, and BWC were significantly reduced, and neurological function was markedly improved compared to the MIS and DFX groups. MIS for intracerebral hematoma removal combined with DFX treatment increased the perihematomal claudin-5 and ZO-1 expression levels, as well as decreased the BBB permeability in rabbits. MIS combined with the DFX strategy may be the ideal option for ICH patients.

In conclusion, MIS combined with DFX treatment could relieve the mechanical compression of brain tissue by the hematoma, significantly reduce iron overload around the hematoma, decrease BBB permeability, alleviate brain edema, and improve neurological function. Our findings could offer a novel strategy for ICH treatment.

## Data Availability Statement

The raw data supporting the conclusions of this article will be made available by the authors, without undue reservation.

## Ethics Statement

The studies involving animals were reviewed and approved by the Animal Ethics Committee of Guizhou Medical University, China.

## Author Contributions

SR, SH, and GW drafted the manuscript, conception, design, and data analysis. LW revised the manuscript for content and analysis. YH and JW supervised or coordinated the experiment. All authors contributed to the article and approved the submitted version.

## Funding

This work was supported by grants from the Science and Technology Foundation of Guizhou Province [No. Qiankehe Foundation (2020) 1Y316], the Cultivation Fund of the Affiliated Hospital of Guizhou Medical University for National Natural Science Foundation of China [No. gyfynsfc (2020)-11], and the Doctoral Research Start-up Fund of the Affiliated Hospital of Guizhou Medical University (No. gyfybsky-2021-30).

## Conflict of Interest

The authors declare that the research was conducted in the absence of any commercial or financial relationships that could be construed as a potential conflict of interest.

## Publisher's Note

All claims expressed in this article are solely those of the authors and do not necessarily represent those of their affiliated organizations, or those of the publisher, the editors and the reviewers. Any product that may be evaluated in this article, or claim that may be made by its manufacturer, is not guaranteed or endorsed by the publisher.
